# Leaderless genes in bacteria: clue to the evolution of translation initiation mechanisms in prokaryotes

**DOI:** 10.1186/1471-2164-12-361

**Published:** 2011-07-12

**Authors:** Xiaobin Zheng, Gang-Qing Hu, Zhen-Su She, Huaiqiu Zhu

**Affiliations:** 1 State Key Laboratory for Turbulence and Complex Systems and Department of Biomedical Engineering, College of Engineering, Peking University, Beijing 100871, China; 2 Center for Theoretical Biology, Peking University, Beijing 100871, China; 3 Center for Protein Science, Peking University, Beijing 100871, China; 4 Current address: Laboratory of Molecular Immunology, National Heart, Lung and Blood Institute, National Institutes of Health, Bethesda, Maryland 20892, USA

## Abstract

**Background:**

Shine-Dalgarno (SD) signal has long been viewed as the dominant translation initiation signal in prokaryotes. Recently, leaderless genes, which lack 5'-untranslated regions (5'-UTR) on their mRNAs, have been shown abundant in archaea. However, current large-scale *in silico *analyses on initiation mechanisms in bacteria are mainly based on the SD-led initiation way, other than the leaderless one. The study of leaderless genes in bacteria remains open, which causes uncertain understanding of translation initiation mechanisms for prokaryotes.

**Results:**

Here, we study signals in translation initiation regions of all genes over 953 bacterial and 72 archaeal genomes, then make an effort to construct an evolutionary scenario in view of leaderless genes in bacteria. With an algorithm designed to identify multi-signal in upstream regions of genes for a genome, we classify all genes into SD-led, TA-led and atypical genes according to the category of the most probable signal in their upstream sequences. Particularly, occurrence of TA-like signals about 10 bp upstream to translation initiation site (TIS) in bacteria most probably means leaderless genes.

**Conclusions:**

Our analysis reveals that leaderless genes are totally widespread, although not dominant, in a variety of bacteria. Especially for *Actinobacteria *and *Deinococcus-Thermus*, more than twenty percent of genes are leaderless. Analyzed in closely related bacterial genomes, our results imply that the change of translation initiation mechanisms, which happens between the genes deriving from a common ancestor, is linearly dependent on the phylogenetic relationship. Analysis on the macroevolution of leaderless genes further shows that the proportion of leaderless genes in bacteria has a decreasing trend in evolution.

## Background

As the first stage of protein synthesis in gene expression, translation is a key process highly conserved in the biological system. Up to now, 31 universally occurring genes identified in 191 species are shown being involved in the translation process [1]. However, translation initiation shows great variation in the three kingdoms. In eukaryotes, the ribosome binds at the 5'-end of the capped mRNA and slides downstream to find the first start codon and then initiate the translation, which is the so-called scanning mechanism [2]. In prokaryotes, there are two known mechanisms. The Shine-Dalgarno (SD) initiation mechanism was found early in *Escherichia coli *[3]. For this mechanism, a short motif called SD sequence in the 5'-untranslated region (5'-UTR) on mRNA binds with the 3'-end of 16S rRNA on the ribosome and helps the ribosome directly identify the translation initiation site (TIS). The other one, namely leaderless initiation, was found later in *λ*-phage of *E. coli *[4]. In this case, the mRNA lacks a 5'-UTR and hence has no SD sequence in it, thus the start codon itself serves as the most important signal for the translation initiation. There were ever propositions that signals downstream of the start codon called "downstream boxes" may bind with the 16S rRNA and help translation initiation of leaderless genes, but these suggestions were then refuted by experimental evidences [5]. In fact, several studies reported that leaderless initiation uses an alternative way which is like that in eukaryotes and the leaderless *λ*cI gene can be faithfully translated *in vitro *in all three kingdoms [6-8]. What is more, this suggests that this initiation way may be the one used by the last universal common ancestor (LUCA) and is conserved in all three kingdoms [2,7,9].

Regarding the diversity of translation initiation mechanism, SD initiation has long been considered the dominant way in prokaryotes. However, recent studies revealed that leaderless initiation should be as important as SD initiation in archaea, one of two branches in prokaryotes. Computational study of 144 genes in the archaeal *Sulfolobus solfataricus *indicated that distal genes in operon are SD-led while single genes and proximal genes in operon are leaderless [10]. Further computational analysis showed that leaderless genes are demonstrated with a rather high proportion in the archaeal *Pyrobaculum aerophilum *[11]. Torarinsson *et al*. also analyzed 18 complete archaeal genome sequences and estimated the number of SD-led genes as well as leaderless genes, their results indicate that at least 12 of 18 archaeal genomes have plenty of leaderless genes [12]. In addition, experimental study in *Pyrobaculum aerophilum *and *Haloarchaea *also reported that the majority of transcripts are leaderless in those archaeal genomes [11,13].

In spite of above-noted efforts to understand on archaea, little is known about the bacteria-wide situation of leaderless genes. One of major reasons is that translation initiation was believed more complex in archaea than in bacteria [12], which led to lack of attention on this issue and continuing to use the canonical explanation by neglecting leaderless initiation mechanism in bacteria. Recently, a few experimental verified leaderless genes were reported in bacteria [5], however it is extremely difficult to explore a clear scenario of the leaderless genes at the bacteria-wide level since them seem to scatter occasionally in some bacterial genomes with a shortage of known data. Nevertheless, there were some computational analyses in respect of the translation initiation mechanism in bacteria, however mainly based on the genes likely using SD-led initiation way, other than the leaderless one. SD sequences in 21 bacterial and 9 archaeal species were investigated and found their occurrence varied from 10.8% to 90.1% [14]. Chang *et al*. also studied 141 bacterial and 21 archaeal complete genomes and gave number estimates of SD-led genes from 11.6% to 90.8% [15]. A most recent work is noteworthy: Nakagawa *et al*. analyzed 277 prokaryotes (249 bacteria and 28 archaea) to survey the proportion of SD-led genes in each genome, and then to discuss the link with initiation mechanism [2]. However, knowing the proportion of SD-led genes does not lead to the knowledge of the leaderless ones. Moreover, to estimate the number of SD-led genes, most of these algorithms usually detected SD signals by a simple scanning method which may ignore the nucleotide composition bias of each genome. At the same time, lack of the test of statistical significance would not give a solid evidence for meaningful signals detected in these algorithms. With currently more than thousand of complete bacterial genomes deposited in the public database or in sequencing, it is more and more significant to reveal the translation initiation mechanism by a clear picture of leaderless genes at the state of the art bacteria-wide level, which should be based on a more accurate and reliable analytical study.

The objective of this study is to answer the question to understand the translation initiation mechanism and its evolutionary scenario in view of leaderless genes in bacteria. We developed an algorithm, which is validated with statistical significance, to classify the initiation regulatory signals upstream to gene start into SD-like, TA-like and atypical signals for all genes in each prokaryotic genome. The method leads to definite identification of both leadered and leaderless genes in a genome. We examined 953 bacterial and 72 archaeal genomes and annotated diverse translation initiation signals in these genomes. Focusing on the leaderless genes and their initiation signals, our analysis reveals that leaderless genes are totally widespread, although not dominant, in a variety of groups in bacteria. What more useful is the quantitative relationships of evolution of initiation signals in bacteria, which might provide a clear picture of the evolution of translation initiation mechanisms.

## Results

**Figure 1:**
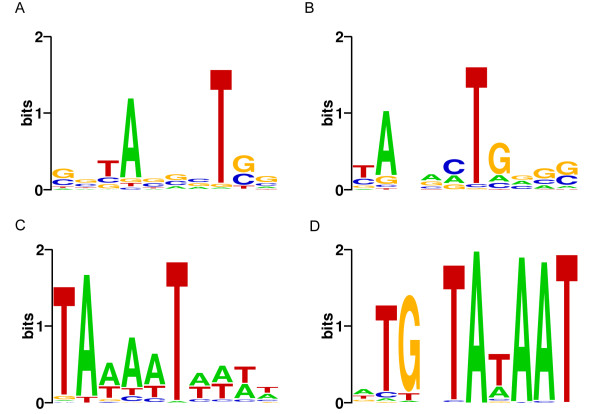
**Sequence logos of TA-like signals in bacteria**. Sequence logo [51] of TA-like signals found in four bacterial genomes: (A) *Streptomyces coelicolor *A3(2) (*Actinobacteria*); (B) *Deinococcus radiodurans *R1 (*Deinococcus-Thermus*); (C) *Aquifex aeolicus *VF5 (*Aquificae*); and (D) *Lactococcus lactis *subsp. lactis Il1403 (*Firmicutes*). The height of a letter on a given position is proportional to its frequency of occurrence. A letter is shown upside-down if the frequency is lower than background.

**Figure 2:**
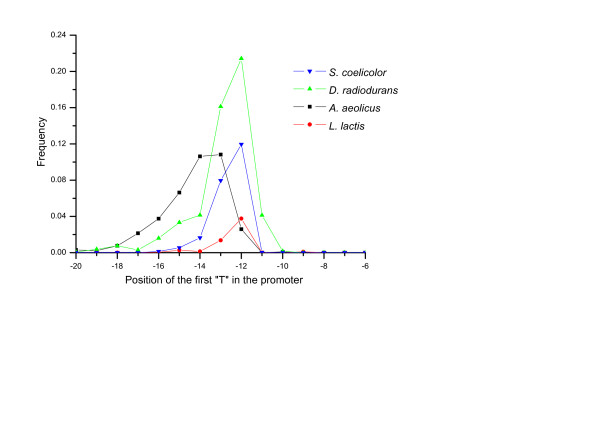
**Positional distribution of TA-like signals to translation initiation site (TIS) in bacteria**. Four bacterial genomes are: *Streptomyces coelicolor *A3(2) (*Actinobacteria*); *Deinococcus radiodurans *R1 (*Deinococcus-Thermus*); *Aquifex aeolicus *VF5 (*Aquificae*); and *Lactococcus lactis *subsp. lactis Il1403 (*Firmicutes*). Y axis shows the frequency that a signal located *X *bps, counted from the first "T" in the "TANNNT" core, upstream to TIS is a Pribnow box. The position of TIS is defined as 0.

We have performed a thorough analysis on 953 bacterial as well as 72 archaeal genomes (TIS upstream sequences 20 bp for bacteria and 50 bp for archaea) by classifying all genes in each genome into categories of being SD-led, TA-led or atypical, meanwhile annotating the translation initiation signal for each gene. The definition and algorithm can be seen in *Materials and methods *for details. The translation initiation signal annotations are available at our webpage [16]. Generally speaking, TA-like signals are found in many genomes including both archaea and bacteria. In archaea, it has been discussed that TA-like signals at around 30 bp upstream to the TIS mean leaderless genes, which use leaderless initiation. While in bacteria, TA-like signals (Figure 1) are identified at around 12 bp upstream to the TIS (Figure 2), and have a consensus of TANNNT, which resembles the -10 box of σ^70 ^factor binding site "TAtaaT" in *E. coli *[17]. These TA-like signals are likely to be transcription promoters and should appear at 10 bp upstream to the transcription start sites (TSSs) [17]. Therefore, the TA-led genes have very short or missing 5'-UTRs and are thus leaderless genes. In brief, our analysis demonstrates that many bacterial genomes have a substantial proportion of leaderless genes. Based on the profile of signals recovered from the more than one thousand genomes, we then report our survey on translation initiation mechanism in the following subsections.

### Validation of the algorithm

**Figure 3:**
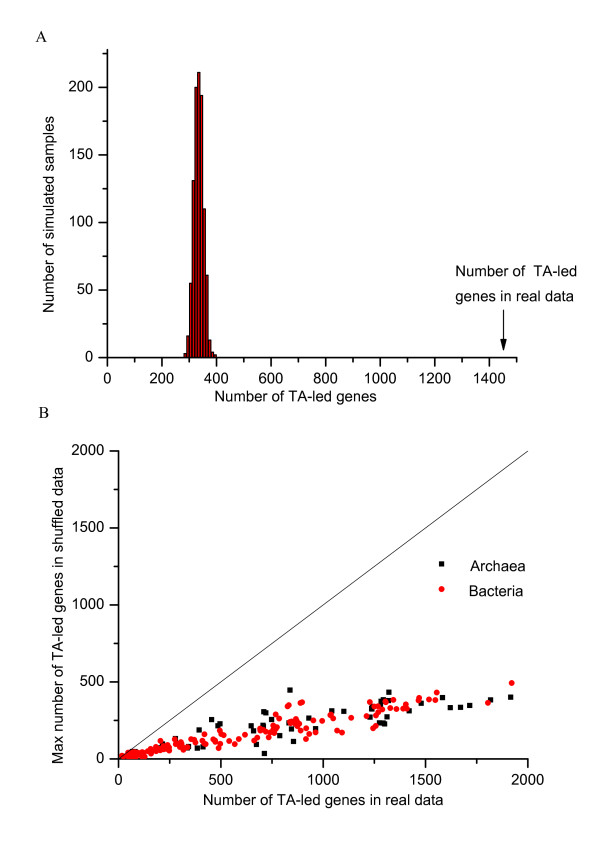
**Validation of the signal detection algorithm**. (A) Distribution of number of TA-like signals identified in randomly shuffled TIS upstream sequences retaining dinucleotide frequency of 7769 genes for the *S. coelicolor *A3(2) genome. (B) Simulative test for the statistical significance of TA-like signals detected by the algorithm. *X *axis is the number of TA-led genes detected in the real data. *Y *axis shows the maximum number of TA-led genes detected in 1000 shuffled datasets. All points are remarkably below the *Y = × *line. All 206 bacterial genomes detected with leaderless genes have been analyzed.

To demonstrate how sure we can be that the relationship between the signals detected through the algorithm and gene translational initiation, we first show evidence for the statistical significance of the TA-like signals found in bacterial genomes, and then estimate the accuracy of our prediction for leaderless genes. A limiting case of examples is the *E. coli*-K12 genome, which is commonly known with leadered genes. For this genome, the algorithm positively detects the SD-like genes and a small quantity of atypical genes. Herein we go on further to make the assessment on strain *Streptomyces coelicolor *A3(2), which belongs to a family of ubiquitous gram-positive soil bacteria used to produce the natural antibiotics, and is also a model organism for the study of leaderless genes [18]. The *S. coelicolor *A3(2) genome has one chromosome and two plasmids. In the chromosome, 20 bp TIS upstream sequences of totally 7769 protein-coding genes are extracted. As a result, among the 7769 genes the gene-classification procedure identifies 1469 genes (18.9%) that have the TA-like signal and they are hence classified as leaderless genes. A shuffling test (See Method for details) shows that no more than 400 TA-led genes would be identified in random sequences retaining dinucleotide frequency (Figure 3A). The validation was also performed on all other genomes in which leaderless genes are detected (as below 59 archaeal genomes, and 206 bacterial genomes detected with leaderless genes in addition to *S. coelicolor *A3(2)), similar results demonstrate that our algorithm is based on a statistical significance (Figure 3B).

A search is further performed in Google Scholar with the key words "leaderless and *Streptomyces coelicolor*" followed by reading the literatures, it reveals only 13 leaderless genes documented for *S. coelicolor *A3(2), of which most of the TSSs are coincident with the first nucleotide of putative TISs. Among the 13 genes, 10 genes (Table 1) have an apparent Pribnow box upstream to their TISs (Other three are *whiH*, with a -10 promoter GCCGACAA recognized by σ*^WhiG ^*factor [19], as well as the genes *ptsH *[20] and *sigJ *[21], which are both likely to be regulated by σ factors other than the σ^70^). Our annotation demonstrates that 9 of these 10 leaderless genes (except *vanH*) are predicted by us (9/10 = 90%), and the predicted Pribnow boxes generally agree with those proposed in the literatures (see Table 1).

**Table 1:** Documented leaderless genes in *S. coelicolor *A3(2)

Gene name	Sequence upstream of TIS^1^	Probability^2^	**Ref**.
*absA*	CGCTCT*TGTAGCGTGC*TGGA**A**TG	0.95	[52]
*devA*	CGAAGT*TGTAGCGTTT*GGTC**G**TG	0.93	[53]
*fabD*	CCTCAA*GAGAGAGTGT*GAGA**G**TG	0.51	[54]
*furA*	CAGGG*AGTAGGTTCG*CCGCC**A**TG	0.80	[55]
*KbpA*	GACGCG*GTTACTTTGA*CGGC**A**TG	0.93	[56]
*malR*	GACA*GGTACAGTCC*ACCCCT**G**TG	0.63	[57]
*phoRP*	GCCGTG*CCTAACCTGG*AGAC**A**TG	0.91	[58]
*vanH*	GAGGCG*CCTTGAATAG*AGGC**A**TG	0.27	[59]
*vanK*	CCGCCG*CCTTGACTGG*GGG**CA**TG	0.78	[59]
*vanR*	CGACT*CGTAATCTCG*ACACC**A**TG	0.85	[59]
*ptsH*	CACCTGAAGGGCAGCA**T**CACATG	0.00	[20]
*sigJ*	GCCTCGGGTAGAAAATCCAC**A**TG	0.03	[21]
*whiH*	TCGGCGCCGACAAAGGAT**GC**GTG	0.09	[19]

^1^The last three characters are triplet for start codons, experimentally determined TSSs are bold, and the most-likely Pribnow boxs are italic. The -10 promoters proposed in literature, if available, are underlined.

^2^The probability that a gene is a leaderless gene regulated by σ^70 ^was calculated by Eq. 5.

It should be noted that the current algorithm is not sensitive enough to the genome when it has a few leaderless genes. In addition, with a widely-used Pribnow box as reference consensus (see *Methods *for details), the algorithm maybe fail in detecting some few leaderless genes with non-Pribnow signal for bacteria in this study. So when a bacterial genome is not judged by the algorithm having leaderless genes, it does not simply mean that the genome has not any leaderless genes, perhaps its leaderless genes are very small in number or without the common Pribnow signal. However in summary, the simulation based on large sample size shows that our TA-like signal prediction has a significant difference from the random strings by the background. This may lead to identifying leaderless genes with a statistical reliability in the current study. Although it is difficult to estimate the prediction accuracy due to lack of experimental data, our prediction is still shown to include most of the known leaderless genes literature-documented at least in this example.

### High usage of leaderless genes in archaea

Based on the leaderless genes identified for each genome, we then present an overall view of leaderless genes in all archaea studied here (see Table 2). On the whole, 59 of 72 sequenced genomes are shown to have leaderless genes, with an average proportion of 38.9%. Among them, 21 of 24 *Crenarchaeal *organisms have averaged 49.5% leaderless genes in their genomes. The three genomes missing leaderless genes are *Desulfurococcus kamchatkensis*, *Hyperthermus butylicus *and *Staphylothermus marinus *respectively (For a list of these Latin names and their relations, see Additional File 1: Table S1). In *Euryarchaeota*, 36 of 46 sequenced genomes have averaged 31.7% leaderless genes. Actually, all euryarchaeal genomes missing leaderless genes belong to Methanogens, which is consistent with the notion that leaderless genes are undetectable in *M. kandleri *and *M. jannaschii *[12]. However, we identified leaderless genes in 12 of the 23 Methanogens. The 12 genomes have an averaged G+C content of 47.2 ± 8.7% while the remaining 11 genomes have G+C content of 34.4 ± 9.0%. This shows that the presence of leaderless genes is related to the G+C content of the genomes. In addition, the two deep branching archaeal genomes, *Korarchaeum cryptofilum *OPF8 and *Nanoarchaeum equitans *Kin4-M, have 41.1% and 71.8% leaderless genes, respectively. Therefore, our analysis well demonstrates the common sense that the archaeal genomes have a high proportion of leaderless genes [10,11], in more detail, with a wide variation from 3.8% to 71.8%.

**Table 2:** Distribution of leaderless genes in sequenced prokaryotic genomes

Group	Number^1^	Avg. Percentage^2 ^(%)
Archaea		
*Crenarchaeota*	21 (/24)	49.5 (6.1, 66.7)
*Euryarchaeota*	36 (/46)	31.7 (3.8, 63.3)
*Nanoarchaeota*	1 (/1)	71.8 (71.8, 71.8)
Other Archaea	1 (/1)	41.2 (41.2,41.2)
Bacteria		
*Acidobacteria*	3 (/3)	13.3 (9.7, 19.4)
*Actinobacteria*	86 (/89)	19.2 (2.3, 29.8)
*Aquificae*	2 (/5)	26.5 (20.7, 32.4)
*Bacteroidetes/Chlorobi*	1 (/37)	2.4 (2.4, 2.4)
*Chloroflexi*	4 (/12)	4.7 (4.0, 5.7)
*Deinococcus-Thermus*	5 (/5)	39.4 (35.9, 46.1)
*Firmicutes*	56 (/209)	4.2 (2.4, 16.6)
*α-Proteobacteria*	2 (/117)	6.3 (4.7, 7.8)
*β-Proteobacteria*	9 (/71)	6.8 (2.8, 10.6)
*γ-Proteobacteria*	15 (/239)	4.5 (3.0, 5.7)
*δ-Proteobacteria*	8 (/33)	15.8 (5.7, 20.6)
*ε-Proteobacteria*	2 (/26)	2.9 (2.7, 3.2)
*Spirochaetes*	6 (/18)	3.9 (1.8, 6.9)
Other Bacteria	8 (/20)	7.8 (1.4, 16.1)

^1 ^The number of genomes having leaderless genes. The numbers in parentheses show how many genomes have been sequenced in these groups.

^2 ^The average percentages of leaderless genes. The numbers in brackets are the minimum and maximum percentages of leaderless genes in these genomes.

### Diverse distribution of leaderless genes in bacteria

It is of great interest in this study to discuss the leaderless genes in bacteria. Below we report the analysis of leaderless genes in all bacterial genomes (see Table 2). Overall, the results show that the algorithm detects 207 among 953 bacterial genomes having leaderless genes, and these 207 genomes include *Acidobacteria*, *Actinobacteria*, *Aquificae*, *Bacteroidetes*/*Chlorobi*, *Chloroflexi*, *Deinococcus*-*Thermus*, *Firmicutes*, *Proteobacteria *(α, β, γ, δ, and ε), *Spirochaetes*, and one unclassified in RefSeq. Unlike in archaea, leaderless genes are not identified in most genomes, however are shown to concentrate in a few groups. The most notable group is *Actinobacteria*, including high-GC and gram-positive genomes. The *Actinobacteria *usually live in a variety of natural environments such as soil, freshwater and the sea, and some of them being human pathogens, meanwhile are well known as secondary metabolite producers and are important in pharmaceutical industry [22]. Due to their importance, 89 species have been completely sequenced. Despite the biodiversity in the 89 genomes, our method detects leaderless genes in nearly all (86 genomes) with proportions around 20%. This is in accordance with previous reports of leaderless genes found in *Streptomyces *and *Corynebacterium *[23]. The *Deinococcus-Thermus *group is also noticeable, although there are only five genomes completely sequenced. Our analysis shows that all five have leaderless genes with high proportions (around 40%). Among them, the *Deinococcus radiodurans *R1 genomes is detected with the highest occurrence of leaderless genes (46.1%) in all bacteria. In fact, this group includes the *Deinococcus *species which are radiation-resistant, and *Thermus thermophilus *which is thermophilic. The high presence of leaderless genes in these genomes may highly probably correspond to their extreme habitat, as suggested for archaea [24]. Besides these two groups, leaderless genes are shown to present in a few genera or species for other groups. For example in γ-Proteobacteria, leaderless genes are most found in *Xanthomonadales *and *Legionellales*, which are located near the root of γ-Proteobacteria, showing that the missing of leaderless genes in other γ-*Proteobacteria *may be due to one loss event during the genome evolution. In *Firmicutes*, leaderless genes concentrate in two clades, *Lactobacillales *and *Mycoplasma*. In *Lactobacillales*, leaderless genes are identified in two subclades: the *Lactococcus*-*Streptococcus *and the *Oenococcus*-*Leuconostoc *clade, while not in *Enterococcus *and *Lactobacillus*. Neither genomes with leaderless genes nor those without leaderless genes form a monophyletic group, probably showing the complex evolution of translation initiation mechanism in *Lactobacillales*. In addition, leaderless genes are found in five genomes in *Mycoplasma*, with an averaged occurrence of 18.3%.

**Figure 4:**
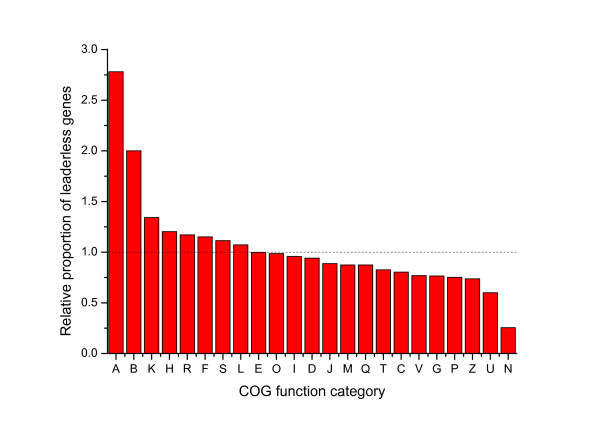
**Relative proportion of leaderless genes in COG function categories**. The function categories are: A, RNA processing and modification (information storage and processing); B, chromatin structure and dynamics(information storage and processing); K, transcription (information storage and processing); H, coenzyme transport and metabolism (metabolism); R, general function prediction only (poorly characterized); F, nucleotide transport and metabolism (metabolism); S, function unknown (poorly characterized); L, replication, recombination, and repair (information storage and processing); E, amino acid transport and metabolism (metabolism); O, posttranslational modification, protein turnover, and chaperones (cellular processes and signaling); I, lipid transport and metabolism (metabolism); D, cell cycle control, cell division, and chromosome partitioning (cellular processes and signaling); J, translation, ribosomal structure, and biogenesis (information storage and processing); M, cell wall/membrane/envelope biogenesis (cellular processes and signaling); Q, secondary metabolite biosynthesis, transport, and catabolism (metabolism); T, signal transduction mechanisms (cellular processes and signaling); C, energy production and conversion (metabolism); V, defense mechanisms (cellular processes and signaling); G, carbohydrate transport and metabolism (metabolism); P, inorganic ion transport and metabolism (metabolism); Z, cytoskeleton (cellular processes and signaling); U, intracellular trafficking, secretion, and vesicular transport (cellular processes and signaling); N, cell motility (cellular processes and signaling).

It has been reported that metabolic-related genes, especially for energy production and conversion, tend to show a higher proportion of SD-containing genes [2]. Here we did a similar study on the COG (cluster of orthologous groups [25]) function categories of leaderless genes in all genomes having leaderless genes. The results show that the information storage and processing categories have relatively higher proportion of leaderless genes (Figure 4). Especially in the COG categories of RNA processing and modification (A) and Chromatin structure and dynamics (B), the proportion of leaderless genes is much higher than in other categories. It is worth noting that these two categories have only a small number of genes in prokaryotes, but are more widespread in Eukaryotes. Leaderless genes are also more likely to present in genes belonging to the COG category of transcription (K), but less likely to present in genes of translation (J). The cellular processes and signaling categories generally have lower proportion of leaderless genes.

As the transcription promoters well as the translation initiation signals for leaderless genes, the TA-like signals detected in bacteria merit a conscientious attention. In fact as shown in Figure 1, our results revealed the variation of the detected TA-like signals across species. In high-GC (> 60%) groups such as *Actinobacteria *and *Deinococcus-Thermus*, the signals have a TAnnnT pattern where the three positions in the middle provide but little information (Figure 1A-B); in medium-GC (40-60%) groups such as *Aquificae *and *Chloroflexi*, the signals have a TAtaaT pattern just like the typical Pribnow box in *E. coli *[17] where the three positions in the middle become more informative (Figure 1C); while in *Firmicutes*, which have mostly low-GC (< 40%) genomes, the signals became TAtAAT, where the two As in the middle became as high as the A and T in the second and sixth positions (Figure 1D). Moreover in several low-GC genomes, the TG peak before Pribnow box can be observed as seen in Figure 1D, which has been reported to substitute for the function of the -35 region [17]. For example in *Lactococcus*, the TG peak is extremely strong and this probably means that the -35 region is missing in this genus. This is probably because the signal is AT-rich, and in low-GC genomes, the signal needs to be strengthened to remain informative, while in high-GC genomes, it degrades weaker as the TAnnnT pattern already provides enough information. Considering our algorithm has been designed to compensate the nucleotide composition bias from genomic background, such a tendency of signal conservation by different GC content seems to be nontrivial and worth further studying. More specifically, this tendency is most probably related with genome evolution in some phylogenetic bacterial groups.

### Atypical genes in prokaryotes

Besides the typical TA-like and the SD-like signals, the algorithm also identifies a lot of so-called atypical signals. Genes bearing these signals, namely atypical genes, are probably SD-less leadered genes, however they could also be leaderless genes with unknown promoter signals. In fact, several clades, for example *Mycoplasma*, *Cyanobacteria *and *Bacteroidetes*, are shown to have a substantial number of atypical genes. It is still uncertain to understand the atypical signals detected in both bacteria and archaea. For example in halophilic archaea, most leadered transcripts have no SD sequence in their leader regions [13], and this genes use new translation initiation mechanisms that are still unknown [26]. However, some atypical signals are shown conserved across species and have conserved position distributions relative to TIS. For instance in *Cyanobacteria*, *Proteobacteria *and other groups, a conserved dipyrimidine is located immediately upstream to the TISs, which is consistent with the findings in the previous study [27]. Another AT-rich signal without strong consensus is found in *Bacteroidetes *and many other groups. These AT-rich signals are suggested to bind ribosomal protein S1 [2] or facilitate translation initiation by affecting mRNA secondary structure [28]. Other atypical signals are likely to be patterns of coding regions, transcription factor binding sites, or other unknown translation or transcription signals. Altogether such signal can serve as a target for biologists to decipher its regulation role by experiments, thus leading to a better understanding of the initiation mechanism.

To sum up as a result, our analysis described above revealed that the translation initiation mechanism in bacteria is far from a simple scenario as previously imaged, and therefore a complex scenario with diversity should be rebuilt. Though SD-led genes are dominant in *Firmicutes*, *Proteobacteria*, and many other groups as previously regarded, leaderless genes are found with high occurrence in many groups such as *Actinobacteria *and *Deinococcus-Thermus*. Moreover, many genomes in *Cyanobacteria *and *Bacteroidetes *use neither SD-led genes nor leaderless genes. The translation initiation mechanisms in those genomes are largely unknown yet to be unravelled. In the following part, we try to understand this diversity from an evolutionary point of view.

### Evolution scenario of translation initiation mechanisms

**Figure 5:**
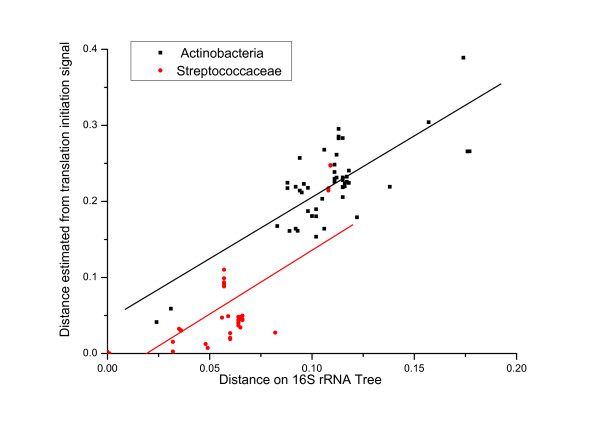
**Substitution rate of translation initiation signals**. The *Y *axis shows the change rate between TA signal and SD signal usage. The *X *axis shows the evolutionary distance of 16S rRNA. Each black point denotes an *Actinobacterial *genome, and all the distances are calculated by comparing with *Streptomyces Coelicolor *A3(2); each red point denotes a genome in *Streptococcaceae*, and the distances are calculated by comparison with *Streptococcus pyogenes *M1 GAS. The linear regressions are *y *= 0.04+1.61*x *(black line, R = 0.77) and *y *= -0.03+1.67*x *(red line, R = 0.77), both with p-value < 0.0001.

It is believed that the mechanism of translation initiation has dynamically changed during the prokaryotic genome evolution [2]. However, it is difficult to speculate an evolutionary history for the translation initiation mechanism even by a rough trend. Herein with a clear classification of initiation signals using by all genes for each genome, it can facilitate exploring the microevolution of translation initiation signals among closely related bacteria. The genome-level distribution of leaderless genes shows that phylogenetically close species tend to be the same behavior of having or having not leaderless genes together. For example, leaderless genes are present in nearly all of the *Actinobacterial *genomes, although they show great variety in their size and habitat. This suggests that the presence of leaderless genes is inheritable and genome-level gains and losses of leaderless genes are nontrivial events in the evolutionary history. To this end, 50 *Actinobacterial *genomes (see our webpage [16] for a list) were compared with the *S. coelicolor *A3(2) genome. For each genome and *S. coelicolor*, orthologous genes are extracted by best reciprocal match. A distance characterizing the substitution of translation initiation signals is then calculated based on Markov model as described in the *Methods *section. It is extremely notable that the distance shows a roughly linear correlation along with the evolution distance of 16S rRNA with a ratio of 1.61 (Figure 5), which corresponds to the substitution rate between SD-like signal and TA-like signal along with the substitution rate of 16S rRNA as species distance for these bacteria. Accordingly in the bacterial genome-level, it implies that the change of translation initiation mechanisms (meanwhile with the change of using translation apparatus), which happens between the genes deriving from a common ancestor, is linearly dependent on the phylogenetic relationship in these species. Moreover, this nontrivial ratio seems to be universal since a similar ratio 1.67 is obtained by comparing 40 genomes in the class *Streptococcaceae *and *Streptococcus pyogenes *M1 GAS (Figure 5).

**Figure 6:**
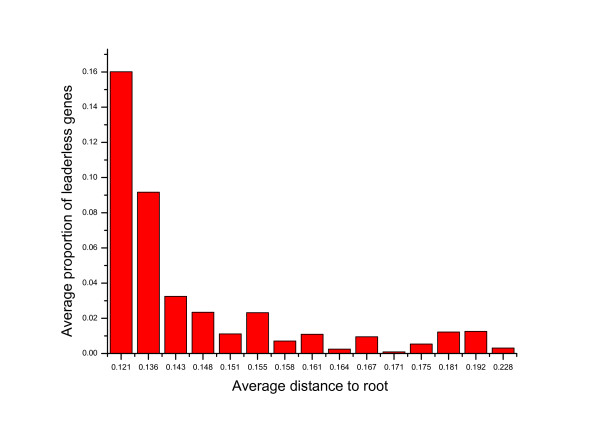
**Trend in the evolution of leaderless genes**. 903 bacterial genomes are put into 15 bins according to their distances to the root (between bacteria and archaea) on 16S rRNA tree. Average proportion of leaderless genes in each bin is calculated. The proportion of leaderless gene shows a rapid decrease followed by fluctuation at a low level.

The distance calculation gives a quantitative description for the substitution rate of translation initiation signals, but it does not give any evolutionary direction. Is there any trend in the macroevolution of translation initiation mechanism? To this end, a tree of the 16S rRNA sequences of 903 bacterial genomes was built and rooted with archaea outgroups. As shown in Figure 6, the 903 genomes were then sorted and put into 15 bins according to their distance to root on the tree. The 60 genomes nearest to root were put into the first bin; the 61-120th nearest were put the second bin, and so on. For genomes in each bin, an average proportion of leaderless gene was calculated. The proportions show a rapid decrease from over 15% to lower than 3% followed by fluctuation around that level. This trend indicates that slower evolving bacteria have more leaderless genes in their genome. If the trend holds, it gives a scenario that the LUCA have even more leaderless genes than 15% in its genome, and leaderless genes have been lost during evolution. Similarly, 64 archaeal genomes were put into two bins according to their distance to root on the 16S rRNA tree. The average proportion of leaderless genes in short-branch genomes is 21.5%, while the number in long-branch genomes is 39.5%. This trend, opposite to that in bacteria, is in accordance with previous report that high levels of leaderless genes predominate in the longer-branch archaea, while organisms with the shortest branches use more SD-led genes [12]. Thus the proportion of leaderless genes in the LUCA might be estimated as between the average level of leaderless genes in short-branch bacteria (about 15%) and the average level of leaderless genes in archaea (about 30%).

**Figure 7:**
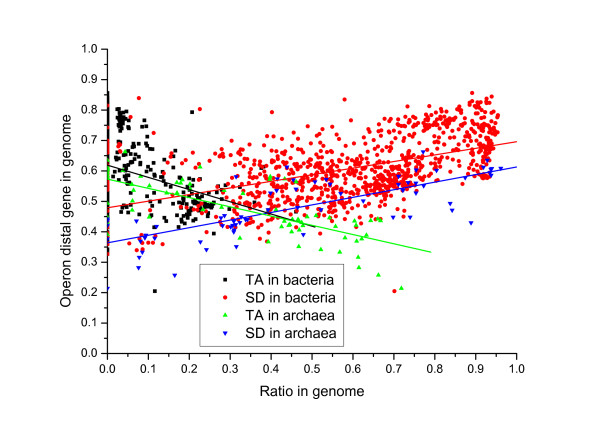
**Coevolution of translation initiation signals and operons**. Each bacterial genome is denoted by two points in the figure (black and red), while each archaeal genome is denoted by a green point and a blue point. The proportion of operon distal genes is positively correlated to SD-led genes while negatively correlated to TA-led genes in both bacteria and archaea. Linear regressions are *y *= 0.61-0.40*x *(black, R = -0.26), *y *= 0.48+0.22*x *(red, R = 0.51), *y *= 0.57-0.30*x *(green, R = -0.73) and *y *= 0.36+0.25*x *(blue, R = 0.76), all p-value < 0.0001.

There is a further question of concern, why leaderless genes were lost in many bacterial genomes while SD-led genes became dominant? One main driving force might be the coevolution of translation initiation signals and the operon structure [24]. Note that in a polycistronic transcript, only the proximal gene could be leaderless while the distal genes usually need SD signals to facilitate translation initiation. If there are a lot of operon distal genes in a genome, there are likely more SD-led genes. For example in *E. coli*, 2704 genes formed 883 operons [29], and the proportion of operon distal genes is (2704-883)/4149 = 43.8%. While in *S. coelicolor *which has more leaderless genes and less SD-led genes, 4416 genes formed 1575 operons [29], and the proportion of operon distal genes is (4416-1575)/7769 = 36.6%, lower than that in *E. coli*. To test this hypothesis, we estimated the proportion of operon distal genes in a genome using gene direction information [30]. The proportion of operon distal genes shows strong positive correlation with the proportion of SD-led genes in both archaea ad bacteria (Figure 7). This suggests that usage of operon structure have strong effect on the evolution of translation initiation mechanism, including the trend of decreasing leaderless genes along with phylogenetic relationship shown in Figure 6. Then why leaderless genes still retain in some genomes? One explanation is that they experienced slower evolution and kept the ancestral features as suggested in archaea [24]. What is more, leaderless initiation also has some possible evolution advantages. One is that it may be used for strict control of some genes so that they would not be translated on spurious transcription of the cognate genes by leakage from upstream operons [31]. Another advantage of leaderless initiation is that it is not affected by some antibiotics that fully inhibit SD initiation [32].

One limitation of the current study should be noticed that only leaderless genes with transcriptional promoters resembling the Pribnow box are identified. This may cause an underestimation for the number of leaderless genes, especially in genomes with a large amount of atypical genes, such as *Mycoplasma*, *Bacteroidetes*, and *Cyanobacteria*. However, σ^70 ^is the most widely used σ factor in bacterial genomes, and its σ_2 _domain is highly conserved with binding to the Pribnow box [33]. It is unlikely that there exist high percentages of leaderless genes not identified by our method. Therefore, despite this limitation, the analysis did suggest the general evolutionary trend of translation initiation mechanisms in bacteria.

## Discussion

Although leaderless genes have been studied extensively in archaea, they have long been regarded as rare events in bacteria [2,24]. Recent works reported the possibility of high occurrence of leaderless genes in some bacterial genomes [2,15], but their propositions are just based on lack of SD sequences in those genomes, and not direct evidence of leaderless genes. In this study, we have clearly shown that leaderless genes are totally widespread, although not dominant, in a variety of groups in bacteria. Two of these groups, *Deinococcus-Thermus *and *Actinobacteria*, deserve serious attention. As the algorithm detects, *Deinococcus-Thermus *is the phylum that has the most leaderless genes, averaged over 30%. It is interesting that most organisms in this group live in extreme environments. For example, *D. radiodurans *is capable of withstanding an acute dose of 5,000 Gy of ionizing radiation with almost no loss of viability [34], while *Thermus *are of the most thermophilic bacteria and can grow at 85°c [35]. These environments may reflect the traits of ancient earth. Thus this suggests an important role of leaderless initiation mechanism playing for the ancestral organisms in the original habitat. The later group *Actinobacteria *is the major antibiotics producer in both nature and industry [22]. It is known that most antibiotics attack the translation system. Specifically speaking, antibiotics such as kasugamycin and pactamycin inhibit translation initiation on leadered transcripts but have no effect on leaderless transcripts[36]. Therefore, the high occurrence of leaderless genes in *Actinobacteria *may suggest the correlation to their antibiotics production.

In our method, all weight matrices and position distributions are learned automatically from the genome sequences. It's worth noting that there are several additional predefined parameters for the model, such as the length of region upstream to TIS in which signals are searched (denoted as *L *in the section Materials and methods). Generally, increasing the length could improve the sensitivity of motif finding. However, this would cost more computation time and the algorithm may find too many unexpected motifs unrelated to translation initiation. Therefore we limited the region to a proper range which is enough for finding signals. In both bacteria and archaea, SD sequences are usually 4-7 bp in length and located at 5-13 bp upstream to the TIS [37], therefore the region of 20 bp upstream TIS is enough to cover the signals. For leaderless genes in bacteria, the -10 box is 6-9 bp in length (TATAAT or with the extended TG) and the distance between the -10 box and the transcription start site varies between -14 and -8 bases (the distance is calculated from the "A" at the second position of the TATAAT box as in the current study [17]). Therefore the -10 box is also covered by the 20 bp region in bacteria. For leaderless genes in archaea, the TATA box is located about 25-37 bp from the transcription start [12] and TIS upstream region of 50 bp is enough to cover it. When longer regions (50 bp upstream to TIS e.g.) are used for searching signals, TA-like signals are found in some new species. However, the position of these TA-like signals are not aggregated and mostly located far from the -10 region of the TIS, and thus these signals are more likely transcription promoters of leadered genes.

According to current evidences, SD-led initiation and leaderless initiation are probably both used by the LUCA. Evidence for the usage of SD-led initiation by the LUCA includes broad usage of SD sequence and the high conservation of anti-SD sequence in both bacteria and archaea [2], and meanwhile leaderless initiation is also proposed to be used by the LUCA considering its usage in all three kingdoms [2,9,24]. However, it is also possible that leaderless initiation originated only in archaea if leaderless gene is a marginal effect in bacteria as long regarded. Our results have shown the broad occurrence of leaderless genes in bacteria, especially in *Actinobacteria*, where around 20% genes are leaderless (Table 2). This indicates that leaderless genes were used by the LUCA, because it is unlikely that the leaderless initiation mechanism originated independently in bacteria and archaea and both become so important. In conclusion, our results provide further support for the proposition that leaderless initiation was used by the LUCA.

## Conclusions

In this paper, we have studied the translation initiation signals in 1025 sequenced prokaryotic genomes and demonstrated the distribution of translation initiation mechanisms used in these genomes. The most surprising finding is that, though not as common as in archaea, there are substantial numbers of leaderless genes in bacteria. Most genomes with high percentage of leaderless genes are located near the root on the 16S rRNA tree and have relatively small number of operon distal genes. These facts show that current leaderless genes in bacteria are likely to be remnants of the ancestor and are retained because those genomes have low demand of organizing genes into operons for highly efficient and specialized gene expression.

## Methods

### Data

953 bacterial and 72 archaeal genomes and their coding gene annotations in this study were downloaded from RefSeq in 2010 [38]. List of the genomes is available at our webpage [16]. To perform analyses of TIS upstream regions, annotations of TISs were collected from the database ProTISA [39] or predicted by TriTISA [40]. ProTISA collects TIS confirmed through a variety of available evidences for prokaryotic genomes, including Swiss-Prot experiments record, literature (IPT), conserved domain hits (CDC) and sequence alignment between orthologous genes (HSC). ProTISA also includes TIS annotations from RefSeq and predicted by TriTISA (MED) [40]. The latest update of ProTISA was released in Oct 2008, corresponding to RefSeq 30. For each gene, we use its TIS in a priority order IPT, CDC, HSC, and MED [39]. Since ProTISA covered only 709 of the 1025 organisms, TISs of the remaining 316 genomes were predicted with TriTISA, which is a TIS predictor with high accuracy [40]. Then for each genome, all TIS upstream sequences were extracted for further analysis. For bacteria 20 bp sequences were extracted, while for archaea 50 bp sequences were extracted since transcription promoters are farther away.

### The multi-signal model and the signal detection algorithm

For the purpose of studying the translational initiation mechanism of prokaryotes, a straight way is to find the sequence patterns in the TIS upstream regions for each genome. With the sequence patterns, an algorithm can then build one or more positional weight matrices (PWMs) to describe the aligned positional frequency corresponding to potential signals. Such a relation has been used in many computational studies of translation initiation mechanism [41-45]. In the current study, based on the similarly motivated strategy we developed a multi-signal model upstream of TIS, and used the Expectation-Maximization (EM) algorithm to estimate the parameters of the model. With the model, we then computationally identified the leaderless genes for each genome.

To detect a signal associated with leaderless genes, the algorithm first selects the search region for each genome and the reference signal consensus. In archaea, leaderless genes can be detected by scanning the reference consensus of TA-rich transcription promoters at about 30 bp upstream to the TISs. Since the promoter mostly occurs at 30 bp upstream to the TSSs, thus for a leaderless gene the TIS and TSS should coincide [10]. In bacteria, the mostly used promoter sequence is the so-called Pribnow box corresponding to the σ^70 ^factor with a T_82_A_89_T_52_A_59_A_49_T_89 _(subscript means the frequency of the nucleotide at that position) pattern and occurs at about 10 bp upstream to the TSS [46]. Thus in this paper, we search the signals in -10 bp regions by using this reference consensus.

#### The multi-signal model of translation initiation signals

Usually the translation initiation signals are conserved in both content and position. For example, in *E. coli*, SD sequences are mostly AGGA, GGAG and GAGG and are within [-10, -5] region to the TIS. Our previous works used a positional weight matrix to characterize the signal content and a discrete distribution to characterize the position [43]. In the current study, motivated by the knowledge of that there may exist three categories of signals, SD sequence, TA-rich transcription promoters and other possible signals in the TIS upstream region for a genome, we generalize the model to describe multiple signals to explore the complexity of the translation initiation mechanism.

Let *S *be the set of *N *sequences of length *L *upstream to TISs. In these sequences, a signal with length *W *can be characterized by a *W *× 4 weight matrix *w*, and each element *w_ib _*of *w *means the probability of base *b *(A, C, G or T) to occur at position *i *in a set of aligned signal representatives. In addition, the signal may occur at any position upstream to the TIS with different probabilities *p_j _*by the distance starting from the *j*-th position of the TIS upstream sequence.

To describe multiple signals, herein we assume that there are *M *signals in the sequence set, but only one in each sequence. We use *w^m ^*and *p^m ^*to denote their weight matrices and positional distributions. For the regions of the sequences not covered by signals, we further build a uniform background model with nucleotide frequency *b*. There is also a probability *p*^0 ^to describe the case that a sequence does not contain any signal and is entirely from the background model.

Denote *Ω *as all the parameters described above, then for each sequence *S_k _*from the set *S*, the probability that it is generated from the model is thus defined by(1)

The likelihood function for the whole set *S *is then written as(2)

We then use the EM algorithm to obtain the maximum-likelihood estimation, details of which are described in Additional File 2: Text S1. As a result for each genome, the EM algorithm based on the multi-signal model is designed to obtain four PWMs as potential signals detected in the genome. It is clear that the more PWMs were searched, the more detailed structures of TIS upstream sequences would be found, but the algorithm will cost much more time and be easier to fall into local maximums. Therefore we select four signals for balance in the algorithm. Other predefined parameters include the sequence length *L*, which is set as 50 for archaea and 20 for bacteria.

#### Classification of translation initiation signals

We then build the links between the four PWMs for each genome and the translation initiation way, which may be used to classify a PWM as which category of signals. A one-by-one manual examining would certainly carry this point. However it is impracticable for large-scale analysis over thousands of PWMs and more than thousand genomes in current study. Therefore, a classifier is designed to computationally calculate each PWM against those widely-known initiation signals as well as the "Atypical" signals [39] for further study.

The first category of signal is the SD sequence widely used in prokaryotes. On 16S rRNA, the sequence UCACCUCCUU near 3'-end is conserved across species [2]. In practice, the motifs AAGG, AGGA, GGAG and GAGG are often used in bacteria, while AGGTG, GGTGA used in archaea [12]. Therefore we use AAGGAGGTGA as a common reference sequence for the SD-like signals, denoted by *R*. For each PWM, let a 5-bp window slide along it meanwhile another along the reference consensus *R*. The distance between the PWM and *R *is calculated as the minimum of the negative log probability that the reference sequence is generated by the PWM, *i.e*.(3)

Our analysis of the distribution of all distances shows that it displays a bimodal distribution separated at approximately 4.0. Then we classify all the PWMs with *SD_dis <*4.0 as SD-like signals.

The second category of signals to be examined is TA signals, which are known to usually appear upstream to the leaderless genes as our main concern in the current study. Since bacteria and archaea use different transcription signals, TA-signals have to be identified separately in two kingdoms. For bacteria, the Pribnow box weight matrix (6 × 4) is calculated by our algorithm from *E. coli *promoter data in PromEC [46] and denoted as *w^r^*. For archaea, the reference weight matrix for TATA-box is calculated from the *Archeoglobus fulgidus *genome (chosen because of its medium GC content). A 6 bp window slides along each PWM and the distance between it and *w^r ^*is calculated as the minimum Euclidian distance as(4)

All the distances also follow a bimodal distribution and the threshold is selected to be 0.8. Therefore the PWMs with TA_dis < 0.8 are regarded as TA-like signals.

In summary, for each PWM from a genome, it is first judged whether can be classified as SD-like signal, then TA-like signal. If neither can be decided, the PWM is regarded as the so-called atypical signal.

#### Classification of genes

Our basic working hypothesis for the model is that each sequence upstream to TIS of a gene has only one or none signal of translation initiation. So it is important to find out which signal each sequence has, this leads to that the gene downstream to the signal is regarded as a leaderless gene or not. As we described in the supporting text, when the model parameters converge,  means the posterior probability that signal *m *appears at the *j*-th position of sequence *k*. When  on all the positions are added together, the posterior probability of signal *m *appearing on sequence *k *can be defined as(5)

meanwhile the probability that sequence *k *does not have a signal is(6)

For each sequence *k*, denote  as the maximum one among  and all , then the sequence may be predicted to have the signal ; if  is the maximum, then the sequence has no signal. As we already classified the signals as SD-like, TA-like and atypical, all the genes are also labeled as SD-led, TA-led and atypical.

### Strategy for validation of the signal detection algorithm

To test the statistical significance of the signals, we use simulated data that retain dinucleotide frequencies of the original sequences. For each 20 bp TIS upstream sequence in a given genome, a corresponding sequence with exactly the same dinucleotide frequency are generated using uShuffle [47]. These simulated sequences form another sequence set and we then apply our gene-classification procedure on it with the real sequence model. The simulation is run 1000 times. If the maximum number of "leaderless genes" identified in shuffled samples is less than the number of "leaderless genes" identified in real data, this means the signal we found is statistically significant with P-value < 0.001.

### Estimation of TIS signal evolution distance

16S rRNA sequences were extracted from RefSeq annotation. After discarding those sequences that are longer than 1600 bp or are shorter than 1400 bp or have too much divergence with other sequences, 967 sequences were left, with 903 bacterial and 64 archaeal genomes. A list of the 967 genomes can be found at our webpage [16]. The sequences were aligned with ClustalW v2.0 [48]. Then a Neighbor-Joining tree was build with Mega 3.1 [49] with Kimura 2-parameter distance model. The tree was rerooted between bacteria and archaea, and distance from each bacterial organism to the root was calculated according to the tree.

For two genomes *A *and *B*, orthologous genes were extracted by finding best reciprocal match of their protein sequences. Denote *N_ll _*as the number of genes that are leaderless in both genomes, *N_ls _*as the number of genes that are leaderless in genome *A *but their orthlogs are SD-led in genome *B*, *N_sl _*as the number of genes that are leaderless in genome *B *but their orthlogs are SD-led in genome *A*, and *N_ss _*as the number of genes that are SD-led in both genomes. Then the distance can be calculated with the logDet distance formula as [50](7)

## Abbreviations

(UTR): Un-translated region; (TIS): translation initiation site; (TSS): transcription start site; (SD): Shine-Dalgarno; (LUCA): Last Universal Common Ancestor; (COG): Clusters of Orthologous Groups.

## Competing interests

The authors declare that they have no competing interests.

## Authors' contributions

XBZ, GQH and HQZ conceived the study, XBZ and GQH designed the data analysis, XBZ and HQZ draft the manuscript, ZSS and HQZ cosupervised the progress of the work. All authors read and approved the final manuscript.

## Supplementary Material

Additional file 1 **All Latin names mentioned in the manuscript and their taxonomic relations**. This additional file is a table listing all Latin names mentioned in the manuscript and their taxonomic relations. Click here for file

Additional file 2 **Details of the EM algorithm to find signals**. This additional file is to describe details of the EM algorithm used in finding translation initiation signals. Click here for file
