# Supplementary material for: Leaderless genes in bacteria: clue to the evolution of translation initiation mechanisms in prokaryotes
Source: BMC Genomics. 2011 Jul 12;12:361. doi: 10.1186/1471-2164-12-361 (PMC3160421; doi:10.1186/1471-2164-12-361)
Supplement: Additional file 2 — Details of the EM algorithm to find signals. This additional file is to describe details of the EM algorithm used in finding translation initiation signals. [file 1471-2164-12-361-S2.DOC]

**Text S1: Maximum-likelihood estimation of model parameters**

We use the EM algorithm to obtain the maximum-likelihood estimation.

1. The algorithm started with random initial parameters *p*, *w* and *b*.
2. For each sequence *S_k_* from the set *S*, each signal and each position, the following posterior probabilities may be calculated as

$\begin{matrix} \lambda_{k}^{0}=\frac{p^{0}\prod_{l=1}^{L} b_{S_{k}[l]}}{P(S_{k}|\Omega)}, & k=1\ldots N \end{matrix}$, (1)

$\begin{matrix} \lambda_{kj}^{m}=\frac{p_{j}^{m}\prod_{l\notin[j,j+W-1]} b_{S_{k}[l]}\prod_{i=1}^{W} w_{iS_{k}[i+j-1]}^{m}}{P(S_{k}|\Omega)}, & k=1\ldots N,m=1\ldots M,j=1\ldots L-W+1 \end{matrix}$. (2)

Here $\lambda_{k}^{0}$ is referred to as the posterior probability that sequence *S_k_* has not signal and $\lambda_{kj}^{m}$ as the posterior probability that signal *m* occurs at the *j*-th position in sequence *S_k_*.

(3) The following intermediate parameters are then calculated:

$\begin{matrix} B_{v}=\sum_{k=1}^{N} \left(\sum_{k=1}^{N} T_{klv}-\sum_{m=1}^{M} \sum_{j=1}^{L-W+1} \sum_{i=1}^{W} \lambda_{kj}^{m}T_{k,i+j-1,v}\right) & v=A,C,G,T \end{matrix}$, (3)

$C^{0}=\sum_{k=1}^{N} \lambda_{k}^{0}$, $\begin{matrix} C_{j}^{m}=\sum_{k=1}^{N} \lambda_{kj}^{m} & m=1\ldots M,j=1\ldots L-W+1 \end{matrix}$, (4)

$\begin{matrix} D_{iv}^{m}=\sum_{k=1}^{N} \sum_{j=1}^{L-W+1} \lambda_{kj}^{m}T_{k,i+j-1,v} & m=1\ldots M,i=1\ldots W,v=A,C,G,T \end{matrix}$. (5)

Here *T* is an indicator variable. *T_klv_*=1 means the *l*-th base on the *k*-th sequence is base *v*, *T_klv_*=0 otherwise.

(4) The model parameters are re-estimated as

$\begin{matrix} b_{v}=B_{v}/\sum_{v=A,C,G,T} B_{v}, & v=A,C,G,T \end{matrix}$, (6)

$p^{0}=C^{0}/\left(C^{0}+\sum_{m=1}^{M} \sum_{j=1}^{L-W+1} C_{j}^{m}\right)$, (7)

$\begin{matrix} p_{j}^{m}=C_{j}^{m}/\left(C^{0}+\sum_{m=1}^{M} \sum_{j=1}^{L-W+1} C_{j}^{m}\right), & m=1\ldots M,j=1\ldots L-W+1 \end{matrix}$, (8)

$\begin{matrix} w_{iv}^{m}=D_{iv}^{m}/\sum_{v=A,C,G,T} D_{iv}^{m}, & m=1\ldots M,i=1\ldots W,v=A,C,G,T \end{matrix}$. (9)

(5) The algorithm then goes to the next iteration until the parameters converge.

The EM algorithm converges definitely, however it may fall into the local maximums, especially for a complex landscape of the likelihood. Fortunately, we may solve this problem by redoing the estimation many times with random initial parameter values. The final estimation is determined by comparing the likelihood values obtained from all iterations.
